# Glucagon-like peptide-1 analogue, liraglutide, in experimental cerebral malaria: implications for the role of oxidative stress in cerebral malaria

**DOI:** 10.1186/s12936-016-1486-0

**Published:** 2016-08-24

**Authors:** Brian DellaValle, Casper Hempel, Trine Staalsoe, Flemming Fryd Johansen, Jørgen Anders Lindholm Kurtzhals

**Affiliations:** 1 Department of Immunology and Microbiology, Centre for Medical Parasitology, University of Copenhagen, Copenhagen, Denmark; 2 Department of Biomedical Sciences, Biotech Research and Innovation Center, Faculty of Health Sciences, University of Copenhagen, Copenhagen, Denmark; 3 Department of Clinical Microbiology, Copenhagen University Hospital, Copenhagen, Denmark

**Keywords:** Oxidative stress, GLP-1, CREB, Cerebral malaria, Plasmodium, Liraglutide, ROS, CM

## Abstract

**Background:**

Cerebral malaria from *Plasmodium falciparum* infection is major cause of death in the tropics. The pathogenesis of the disease is complex and the contribution of reactive oxygen and nitrogen species (ROS/RNS) in the brain is incompletely understood. Insulinotropic glucagon-like peptide-1 (GLP-1) mimetics have potent neuroprotective effects in animal models of neuropathology associated with ROS/RNS dysfunction. This study investigates the effect of the GLP-1 analogue, liraglutide against the clinical outcome of experimental cerebral malaria (ECM) and *Plasmodium falciparum* growth. Furthermore the role of oxidative stress on ECM pathogenesis is evaluated.

**Methods:**

ECM was induced in *Plasmodium berghei* ANKA-infected C57Bl/6j mice. Infected Balb/c (non-cerebral malaria) and uninfected C57Bl/6j mice were included as controls. Mice were treated twice-daily with vehicle or liraglutide (200 μg/kg). ROS/RNS were quantified with in vivo imaging and further analyzed ex vivo. Brains were assayed for cAMP, activation of cAMP response element binding protein (CREB) and nitrate/nitrite. *Plasmodium falciparum* was cultivated in vitro with increasing doses of liraglutide and growth and metabolism were quantified.

**Results:**

The development and progression of ECM was not affected by liraglutide. Indeed, although ROS/RNS were increased in peripheral organs, ROS/RNS generation was not present in the brain. Interestingly, CREB was activated in the ECM brain and may protect against ROS/RNS stress. Parasite growth was not adversely affected by liraglutide in mice or in *P. falciparum* cultures indicating safety should not be a concern in type-II diabetics in endemic regions.

**Conclusions:**

Despite the breadth of models where GLP-1 is neuroprotective, ECM was not affected by liraglutide providing important insight into the pathogenesis of ECM. Furthermore, ECM does not induce excess ROS/RNS in the brain potentially associated with activation of the CREB system.

## Background

The *Plasmodium* parasite is estimated to infect hundreds of millions worldwide carrying an annual death toll of approximately 0.5 million [1]. Cerebral malaria (CM) constitutes a large portion of the mortality burden. The pathogenesis of CM is complex involving a vascular component with parasite-induced venule blockage, platelet activation, blood–brain barrier (BBB) disruption and an inflammatory component with activation of astrocytes, microglia, complement system, and lymphocytes. Ultimately; the consequences of this dysfunction can be seizures, coma and death [2–4]. Oxidative stress is present throughout the body from the endogenous immune response and the presence of reactive free haem from lysis of infected erythrocytes (iRBC) [5–7]. Interestingly, although oxidative stress is present systemically, it has been suggested that neither cerebral mitochondrial performance [8, 9] nor antioxidant buffering [10–13] seems decisively compromised in fatal murine and human CM.

Glucagon-like peptide-1 (GLP-1), recently emerging as a front-line treatment for type II diabetes mellitus (DM), has been shown to have potent neuroprotective properties. GLP-1 receptor agonist treatment improves outcomes in experimental models of stroke, Parkinson’s disease and Alzheimer’s disease [14–16]. Moreover, the GLP-1 analogue, liraglutide, is neuroprotective against murine traumatic brain injury (TBI) and this effect is closely associated with the cytoprotective cAMP response element binding protein (CREB) pathway [17]. In addition to the potential cytoprotective effects of CREB-driven protective proteins, GLP-1 has also been suggested to promote endothelial cell nitric oxide production, up-regulate haem oxygenase-1 [18, 19], and reduce neuroinflammation in vivo [20–23], all previously reported to protect against experimental CM (ECM) [7, 20, 24]. The broad spectrum of neuroprotective effects reported after GLP-1 receptor agonist treatment and its safety profile in humans led us to investigate its potential as a therapeutic treatment against murine CM. In this investigation, this therapeutic potential is assessed with the GLP-1 receptor agonist, liraglutide: a long-acting GLP-1 analogue designed to extend the half-life of GLP-1 receptor activation [25] that can cross the BBB [26].

Finally, the prevalence of obesity and type II DM is rising in malaria endemic regions of low and middle-income countries [27] and therefore the safety of liraglutide treatment on parasite growth and metabolism was evaluated.

## Methods

### Liraglutide treatment of *Plasmodium berghei*-induced cerebral malaria

All animal experiments were approved by the Danish Animal Experiments Inspectorate according to the license 2012-15-2934-00449. Survival study experiments were designed from a power analysis that suggested 14 mice per group would be powered to 80 % at 95 % confidence for detecting a 50 % reduction in mortality. Animals were kept under standard conditions with food/water access ad libitum, and all studies were conducted to minimize suffering and in accordance with a pre-defined humane endpoint of body temperature <32 °C [28].

Stock *Plasmodium berghei* strain ANKA parasites characterized previously for disease progression were thawed and injected into a pilot mouse. After 4 days of in vivo growth, 10^4^ iRBCs were injected intra peritoneal (i.p.) into female C57Bl/6j mice for modeling ECM or female Balb/c mice for modeling non-cerebral malaria (NCM) [(Taconic, Denmark), age 6–8 weeks]. To evaluate the potential neuroprotective effects of liraglutide in ECM, liraglutide was administered at 200 μg/kg twice-daily. Initial pilot experiments in ECM were performed with 50, 200, 400 μg/kg and obtained similar results. In previous work, three increasing doses of liraglutide (100, 200, and 400 μg/kg) were investigated as a protective agent in a model of murine TBI [17] where 200 μg/kg was the most effective against brain damage. Moreover, this dose is matched to the dosing in the clinic for anti-diabetic effect in mice [29]. Thus, high-powered experiments were performed at a dose of 200 μg/kg. Maintaining a translational focus in the study, liraglutide was administered from day 4 post-infection to mimic a critical care scenario: day 4 coincides with detection of parasitaemia, and the beginning of damage to the olfactory bulb [30].

Thus, on day 4 post-infection mice were randomly assigned to different treatment arms: twice-daily vehicle s.c. (PBS; n = 15), twice-daily liraglutide s.c. (Novo Nordisk, DK; n = 15), or once-daily 5000 IU recombinant human Erythropoietin i.p. (Epo, Eprex^®^, Janssen-Cilag, Switzerland; n = 8). Epo treatment from day 4 completely protects from ECM development [8, 31] and was therefore included as a positive control group. This ECM study was repeated to confirm results with smaller sample sizes [n = 7 (vehicle), 8 (liraglutide)]. NCM mice were included into evaluate the interaction between liraglutide and parasite growth and to control for the cerebral component of the *P. berghei* infection. These two treatment arms (vehicle and liraglutide) were included in an experiment with ECM and NCM mice (n = 10 for all groups and treatments).

Mice were monitored twice daily on day 4 and three times daily from day 5. 2 μL of blood from the tail vein were sampled from day 5 for parasitaemia enumeration. Parasites were enumerated with flow cytometry using acridine orange labeling and FACScanto flow cytometer (BD Biosciences, CA, US) as previously described [32].

ECM progression was monitored based on presentation of ruffled fur, ataxia, hemiplegia, seizures, coma and core body temperature reduction [28, 33]. This study was conducted with a pre-defined humane endpoint: animals were deemed ‘terminal’ when they registered a body temperature below 32 °C. Body temperature was measured three-times daily, non-invasively with an infrared thermometer (Testo 845, Testo, Germany) and further confirmed with rectal probe (DM852, Ellab, DK) if the measurement was below 32 °C as described in [34]. Mice were killed by cervical dislocation and brain was observed after removal of skull for presence of haemorrhage to further verify the diagnosis of ECM. Finally, a group of healthy C57Bl/6j mice were included and treated from day 4 until day 8 with vehicle or liraglutide (n = 8, 8).

### Reactive oxygen/nitrogen species visualization

In order to visualize ROS/RNS species generation in CM infection in real-time, the highly sensitive luminol derivative, L-012 was used [35]. *Plasmodium berghei*-infected mice were injected with 75 mg/kg L-012 i.p. from day 5 post infection. Mice were anesthetized with isoflurane and chemiluminescence was imaged (dorsal orientation) with a CCD camera 15 min after injection with a 60 s exposure. Thereafter a supine acquisition was taken (60 s exposure). In vivo images were acquired once daily thereafter. Animals that were deemed terminally ill were injected with the luminescent probe and cervically dislocated 15 min post-injection. Organs were rapidly exposed for light emission and an ex vivo image was acquired (60 s). Epo prevented ECM in all mice and thus were terminated at the end of the experiment (day 9) along with uninfected mice for imaging of the organs. In a repeat experiment, *P. berghei*-infected mice were administered vehicle from day 4 and ex vivo tissue images were acquired in mice deemed terminal. Images were quantified with Living Image software (Perkin-Elmer, USA). All quantifications are reported as the difference between regions of interest and background signal.

### Cerebral CREB, cAMP, and nitrate/nitrite levels

Brains were removed, split into olfactory bulb, cerebellum, and two cerebral hemispheres, snap frozen in liquid nitrogen and maintained at −80 °C. One hemisphere was designated for immunoblotting and nitrate/nitrite assay and was homogenized in PhosphoSafe extraction reagent (catalogue number 71296, Millipore, DK) with protease inhibitor cocktail (mini complete, Roche, DK), protein content quantified (BioRad DC protein assay kit, DK), and stored at −22 °C.

One hemisphere was processed and assayed for cAMP determination as per manufacturers recommendations (Enzo Life Sciences, DK) in hydrochloric acid.

Immunoblotting was optimized and performed with standard western blot principles. All homogenates were thawed and mixed in sample buffer (100 mM DTT, Laemmli buffer) and heated at 95 °C for 5 min. Based on optimization experiments, 25 µg protein per animal from one cerebral hemisphere was loaded into pre-cast polyacrylamide gels (12 % bis–tris; NuPAGE, Life Technologies, DK) and gels run in MES buffer at 150 V for 60 min. Gels were transferred to PVDF membranes (BioRad) at 30 V for 60 min. Membranes were then washed in TBS and placed in blocking solution (5 % skim milk powder—(CREB) or bovine serum albumin- (phospho-CREB) TBS-Tween20 (TBST) for 1 h at room temperature. Primary antibodies were applied CREB (1:1000, Cell Signaling, 9197), pCREB (Serine 133) (1:1000, Millipore, 06-519), and GAPDH (1:10 000 Millipore, MAB 374) in appropriate blocking solution overnight at 4 °C. Membranes were washed three times in TBST and secondary antibodies were applied in appropriate blocking solution: HRP-conjugated anti-rabbit/anti-mouse (DAKO, DK) at 1:2000 and 1:3000 respectively for 1 h at room temperature. Membranes were washed three times in TBST and once in TBS. Membranes were incubated in SuperSignal Femto substrate (34095, Fisher, DK) and exposed with CCD camera (Bio-Rad Chemidoc XRS imager). Images were quantified with ImageJ software [36]. All protein signals are reported relative to the housekeeping protein, GAPDH.

Nitrate/nitrite assay was performed as per manufacturer’s recommendations (Cayman Chemical, DK) and normalized to protein content.

### *Plasmodium falciparum* growth and metabolism

Stock *P. falciparum* parasites of CSA-selected Palo Alto (PA) strain were chosen to investigate the effects of liraglutide on parasite growth based on previous growth criteria as described in detail in [37]. Parasites maintained at the Centre for Medical Parasitology were thawed and cultured at 37 °C in Albumax-enriched, HEPES buffered RPMI culture media: RPMI (Gibco, DK), albumax (Gibco), hypoxanthine (Sigma-Aldrich, DK), and gentamicin (Gibco) [37]. Stock cultures were diluted to 0.5 % and cultured in sterile 96-well microplates in vehicle (PBS) or increasing doses of liraglutide (0.1–100 μg/mL). Artesunate (10 ng/mL, Sigma-Aldrich) was included as a positive control for growth inhibition. Media and treatments were removed and replenished after 24 h and parasitaemia quantified by flow cytometry [32]. In a repeat experiment, *P. falciparum* parasites were cultured in sterile 24-well plates with the same treatment groups. Media and drugs were exchanged and replenished after 24 h. After 48 h, media was removed, spun at 4 °C and the supernatant frozen at −80 °C for metabolite quantification.

### Metabolite assay

Media from 24-well cultures were thawed and fed into a blood-gas and metabolite analyzer (ABL 725 Analyzer, Radiometer, DK) as described previously [37]. Media were assayed for concentration of glucose, lactate, H^+^ (pH), Na^+^, K^+^, and Ca^2+^.

### Data analysis

Data sets were tested for normality (Shapiro–Wilk) and equal variance (Barlett’s) before statistical analyses were performed. Non-normal data sets were log-transformed, and tested for normality. All data sets were either normal or normalized after log-transformation and thereafter analysis was performed with parametric tests. Survival data were analysed with log-rank test of Kaplan–Meier curves. One-way ANOVA with Holm-Sidak correction was performed on ex vivo L-012 chemiluminescence data, immunoblotting, cAMP and nitrate/nitrite assays, and cultured-parasite growth and metabolism data. Two-way ANOVA repeated measures was performed on in vivo parasitaemia data and in vivo L-012 chemiluminescence data. Normal data is presented as mean + SEM. and log transformed data as geometric mean + 95 % confidence intervals unless otherwise stated. Significant differences are reported with p values below 0.05. Statistical analyses were performed with Graphpad Prism 6 software.

## Results

### Liraglutide treatment in development and progression of ECM and NCM

**Fig. 1:**
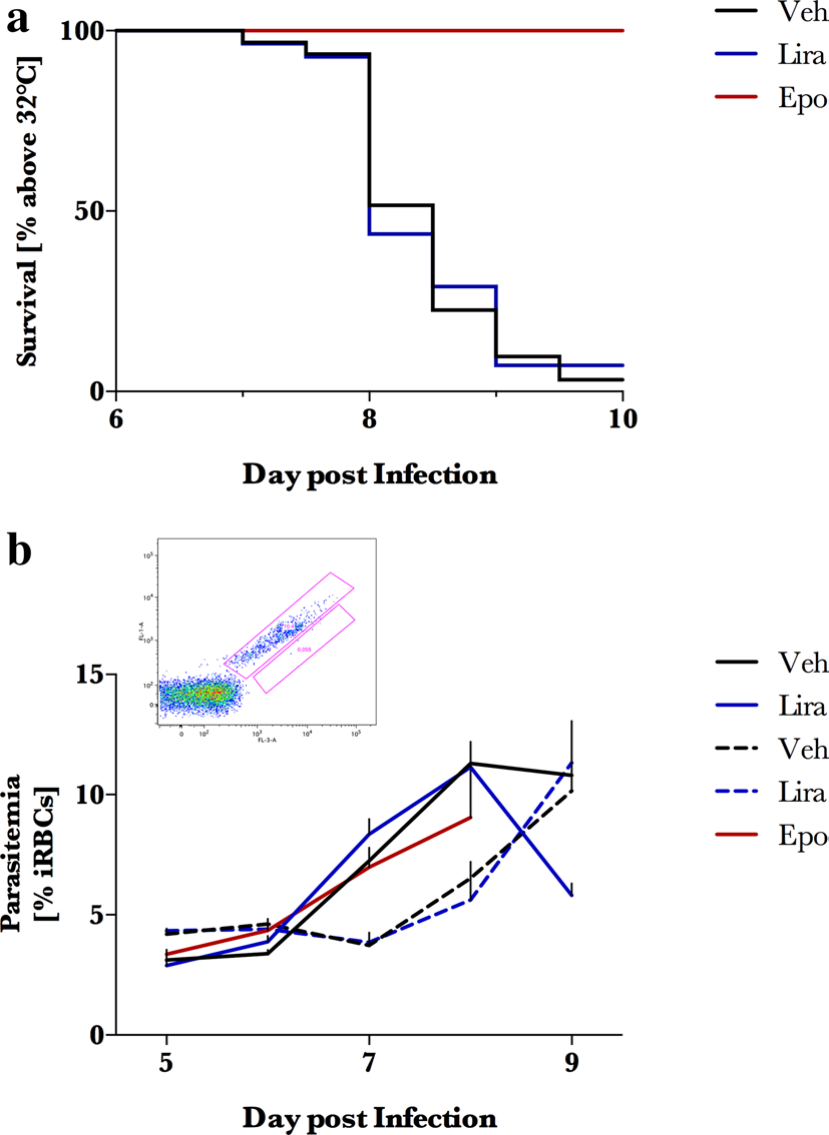
Effects of liraglutide and erythropoietin on parasite growth and animal survival after *Plasmodium berghei* ANKA infection. *Plasmodium berghei* ANKA infected C57Bl/6j [experimental cerebral malaria (ECM; *closed line*)] or Balb/c [non-cerebral malaria (NCM; *dotted line*)] mice were administered vehicle (*black*) or liraglutide (Lira: 200 μg/kg; *blue*) from day 4 post infection. Finally, a group of ECM mice was administered erythropoietin (*red*, Epo) from day 4 post infection. **a** Survival in ECM mice refers to a pre-determined humane endpoint where the animal is deemed terminal when the body temperature is <32 °C. Survival data is presented as a Kaplan–Meier plot. Parasitaemia was enumerated by flow cytometry as described in detail in [32]. Reticulocytes were separated from infected erythrocytes by gating reticulocytes from uninfected control animals. Parasitaemia is presented as line graphs + SEM. All data in **a** and ECM animals in **b** are the combined data from three separate experiments (n = 32, 33). NCM (n = 10, 10) and Epo (n = 8) mice are the results from one experiment

Based on the neuroprotective effects of liraglutide in murine TBI [17], the effects of twice-daily liraglutide at 200 μg/kg were investigated. The vast majority of vehicle—(31 of 32) and liraglutide (31 of 33)—treated mice developed moribund clinical presentation, major body temperature reduction (<32 °C) and macroscopic cerebral haemorrhage. Epo treatment protected all mice from clinical signs of ECM, and macroscopic cerebral haemorrhage. Liraglutide treatment did not affect ECM progression (Fig. 1). Moreover, parasite growth in ECM animals and NCM was not affected by liraglutide treatment (Fig. 1b). In the first experiment with liraglutide treatment, parasitaemia was slightly higher in liraglutide-treated animals. This was not the case in the subsequent two experiments.

### In vivo imaging in ECM: reactive oxygen/nitrogen species generation is high in ECM mice, but is absent in the terminal ECM brain

**Fig. 2:**
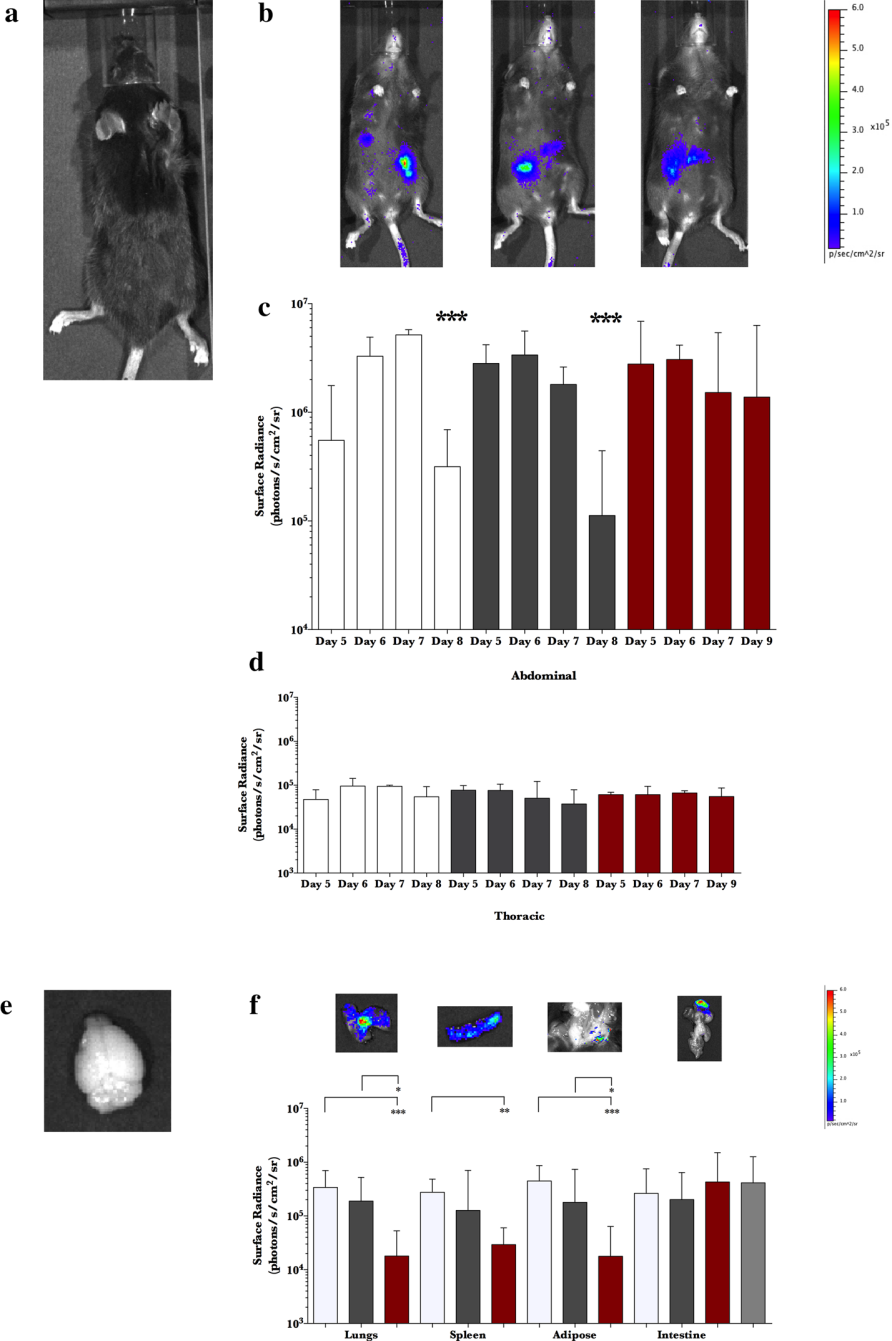
In vivo and ex vivo visualization and quantification of ROS/RNS in *Plasmodium berghei*-infected mice treated with liraglutide and erythropoietin. Reactive oxygen and nitrogen species (ROS/RNS) were visualized and quantified in experimental cerebral malaria. Animals were administered vehicle (*white*), liraglutide (Lira; *black*), or Epo (*red*) from day 4 of *P. berghei* infection. ROS/RNS probe, L-012, was administered at 75 mg/mL i.p. from day 5 post infection and mice were imaged 15 min post injection for chemiluminescence under isoflurane anesthesia. Animals were imaged [dorsal (**a**) and supine (**b**)] daily (photos provided are from day 7). In vivo images were quantified in regions of interest [abdomen (**c**), thorax (**d**)] and presented as difference from background. Animals presented in **b** are representative of day 7 in each treatment group. Animals deemed terminal through humane endpoints described above (i.e. body temperature <32 °C) were injected with L-012. 15 min post-injection, organs were removed, imaged ex vivo for chemiluminescence and reported background-corrected. **e** Brains emitted no signal above background in all terminal mice regardless of treatment (n = 27) nor in Epo treated mice. **f** Lung, spleen, adipose, intestine chemiluminescence from infected animals (vehicle n = 19, 19, 18, 19; Lira n = 8, 7, 4, 8; Epo n = 5, 7, 5, 7) and uninfected controls (*gray*, n = 4). Data was analyzed after log-transformation and presented as *bar graphs* of median + interquartile range on a log10* y*-axis. Significant differences are reported when p values were <*0.05, **0.01, ***0.001

Mice from all three infected groups were followed with in vivo imaging for ROS/RNS generation from day 5 post-infection. Chemiluminescent signals were detected in the lower abdomen from day 5. Interestingly, no signal was detected in the brains of any mice above background signal throughout the development of ECM despite neurological symptoms and cerebral haemorrhage (Fig. 2a). From day 5, L-012 signal from abdomen of vehicle-treated mice tended to increase until day 8 where mice deemed-terminal (i.e. body temperature <32 °C) had significantly reduced abdominal signal (p < 0.05–0.001) (Fig. 2b, c). A similar phenomenon was observed in liraglutide mice in the terminal phase (p < 0.001). Epo-treated mice emitted a similar abdominal signal as vehicle and liraglutide-treated mice from day 5 but did not experience the marked reduction in abdominal signal at day 8 seen in the other groups (change in median from day 7: 81 % (vehicle), 93 % (liraglutide) reduction; Epo: no change (p = 0.9). In the thoracic region, there was no significant fluctuation over time and the signal was not affected by treatments (Fig. 2b, d).

### Ex vivo quantification of ROS/RNS in brain, lungs, spleen, adipose and intestine of mice with fulminant ECM

Tissues were dissected from terminal mice (i.e. body temperature <32 °C) and imaged for chemiluminescence. No signal above background threshold was detected in whole brains or when sliced in a brain matrix in any mice (Fig. 2e). ROS/RNS generation was detected in the lungs, spleen, adipose tissue and intestine of terminal ECM (Fig. 2f) and Epo-treated mice. Uninfected mice did not emit chemiluminescence in the adipose, lungs or brain tissue but developed a signal in the intestinal tract and spleen in 2 of 5 mice (Fig. 2f). Liraglutide treatment did not significantly affect the ROS/RNS generation in lungs, spleen, adipose, or intestine. Curative Epo treatment significantly reduced ROS/RNS in the lungs (p < 0.001), spleen (p < 0.01) and adipose tissue (p < 0.001) compared to vehicle. Intestinal ROS/RNS generation was similar for all groups (p = 0.36). These measurements were repeated in vehicle-treated mice injected from day 4 (n = 7) in a subsequent study. Indeed, no signal was detected in the brain of terminal mice despite severe neurological symptoms and cerebral haemorrhage. Chemiluminescence was detected in lungs, spleen, adipose and intestinal tissue.

### Activation of CREB in ECM and the effects of liraglutide on CREB, cAMP and nitric oxide derivatives

**Fig. 3:**
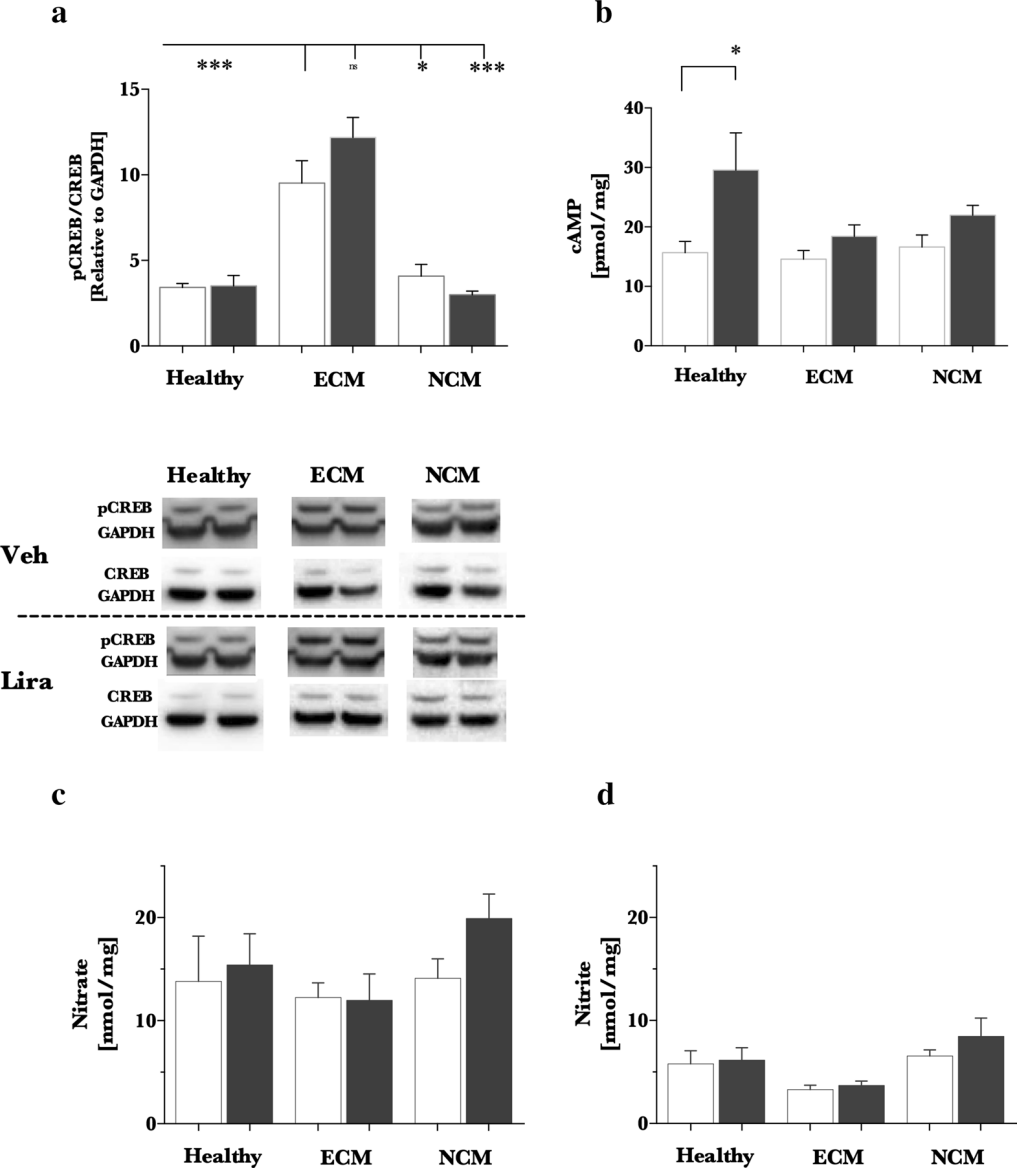
Cerebral CREB, cAMP and nitrate/nitrite levels in *Plasmodium berghei* infection. Animals were administered vehicle (*white*) or liraglutide (Lira; *gray*) twice daily from day 4 post infection in C57Bl/6j mice for the experimental cerebral malaria (ECM) model and Balb/c mice for non-cerebral malaria (NCM) model. Healthy C57Bl/6j mice were treated with vehicle or liraglutide from day 4 to 8 of the experimental period. Brains were excised from terminal mice, the cerebrum isolated, split into two hemispheres, and flash frozen. One hemisphere was homogenized with phosphatase inhibition (Healthy: vehicle n = 7, Lira n = 6; ECM: vehicle n = 5, Lira n = 7; NCM: vehicle n = 6, Lira n = 6) and the other in a hydrochloric acid buffer for cAMP (Healthy: vehicle n = 7, Lira n = 7; ECM: vehicle n = 5, Lira n = 7; NCM: vehicle n = 7, Lira n = 7). Homogenates for immunoblotting were separated by SDS-PAGE and probed for CREB, pCREB_ser133_ and normalized to GAPDH. Each antigen is normalized to GAPDH and presented as a pCREB/CREB ratio (**a**). Cerebral cAMP levels (**b**) and nitrate, nitrite levels (**c**, **d**; Healthy: vehicle n = 7, Lira n = 5; ECM: vehicle n = 4, Lira n = 5; NCM: vehicle n = 5, Lira n = 7) were assayed according to manufacturer’s recommendations. Data is presented as a *bar graph* with mean + SEM

In a previous investigation in TBI, liraglutide treatment markedly increased CREB activation and was protective against ROS/RNS accumulation and mitochondrial dysfunction [17]. As shown in Fig. 3a, CREB activation increased twofold (p < 0.001) with ECM when compared to healthy (p < 0.001) and NCM (p < 0.05) control mice. Nevertheless, liraglutide treatment did not increase activation of CREB in healthy, ECM, or NCM mice when compared to vehicle. Interestingly, liraglutide treatment did increase cAMP levels in the brains of healthy mice (twofold, p < 0.05) but did not increase cAMP in diseased ECM or NCM mice (Fig. 3b). Nitrate and nitrite levels were assessed in the brain to evaluate markers downstream of nitric oxide production. There was no variation across the different groups of mice or between treatment arms in levels of cerebral nitrate or nitrite (Fig. 3c).

### Effect of liraglutide on in vitro growth of *Plasmodium falciparum*

**Fig. 4:**
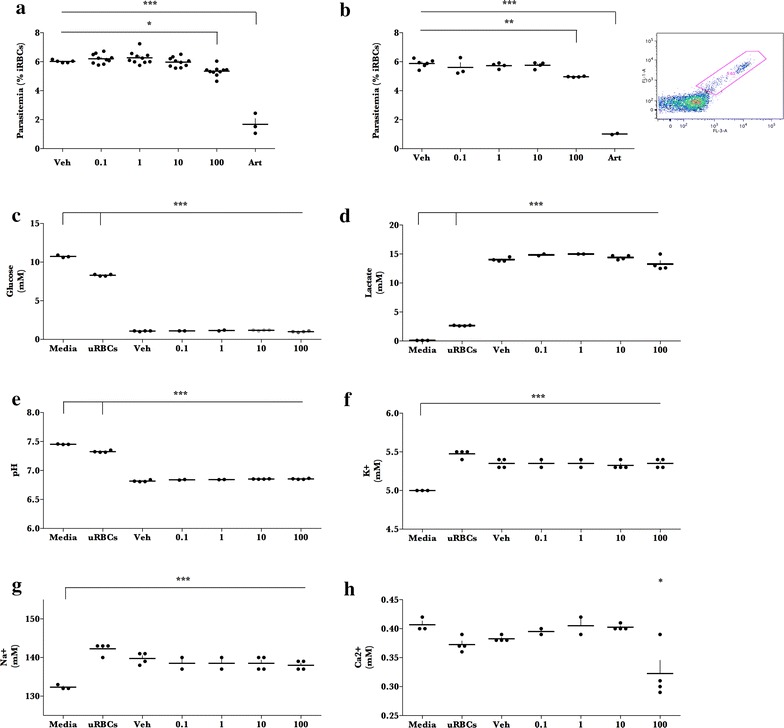
Liraglutide has minor effects on *Plasmodium falciparum* growth and metabolism in vitro. *Plasmodium falciparum* parasites were cultured for 48 h in the presence of increasing doses of liraglutide (Lira: 0.1–100 μg/mL) in **a** 96-well plates for parasitaemia measurements or **b**–**h** 24-well plates for metabolite measurements. Media including drug doses were exchanged after 24 h. Parasitaemia was enumerated with flow cytometry and metabolites were measured in ABL. Data is presented as* dot plot* with mean + SEM (96-well cultures (**a**): vehicle n = 5, Lira 0.1–100 ng/mL: n = 10, Artesunate n = 3; 24-well cultures (**b**); vehicle n = 6, Lira 0.1 ng/mL: n = 3, Lira 1–100 ng/mL: n = 4, Artesunate n = 2; Metabolism (**c**–**h**: Media: n = 3, uninfected erythrocytes (uRBCs): n = 4; vehicle: n = 4; Lira 0.1, 1 ng/mL: n = 2; Lira 10–100 ng/mL: n = 4)

The results from the initial experiment concerning an effect of liraglutide treatment on parasite growth suggested an investigation into the effect of liraglutide on the human malaria parasite, *P. falciparum*, although there was no effect of liraglutide in subsequent experiments. *Plasmodium falciparum* grew efficiently in 96-well plates in vitro in the presence of vehicle (PBS) after 48 h (~6.0 % parasitaemia; Fig. 4a; n = 5 distinct cultures) and artesunate inhibited parasite growth (n = 3). Incubation of *P. falciparum* with liraglutide 0.1–10 μg/mL did not affect parasite growth after 48 h (n = 10 cultures/dosage; p > 0.6). Liraglutide 100 μg/mL reduced in vitro *P. falciparum* growth by 10 % (n = 10, p < 0.05). In a second repeat experiment in 24-well plates, vehicle-treated cultures grew to 5.9 % parasitaemia (48 h growth, Fig. 4b, n = 6 cultures) and liraglutide doses 0.1–10 μg/mL did not affect parasite growth (n = 3–4, p = 0.7–0.9) after 48 h. Liraglutide 100 μg/mL significantly reduced parasite growth by 16 % (n = 4, p < 0.01). Analysis of the media after 48 h growth showed that iRBCs in all groups consumed glucose and produced lactate to the same levels (Fig. 4c, d). Furthermore, levels of H^+^, K^+^, and Na^+^ in growth medium were similar in all groups (Fig. 4e–g). Interestingly, [Ca^2+^] was significantly reduced in the media from cultures with liraglutide treatment at 100 μg/mL (p < 0.05) (Fig. 4h).

## Discussion

Despite the premise for a therapeutic effect of GLP-1 receptor agonism against ECM cerebrovascular pathology these results indicate that liraglutide does not protect against ECM. After pilot testing of various doses and rigourous experimentation with three high-powered studies (Fig. 1a), these data suggest that the finding presented here is robust for this analogue. With the scope of neuropathological conditions that GLP-1 treatment improves [14–17], this finding is insightful for understanding both the pathogenesis of ECM and the neuroprotective mechanism of GLP-1 receptor agonism.

*Plasmodium berghei* ANKA infection initiates an oxidative stress component from rupturing of RBCs releasing free haem, hemozoin and immunogens and activated phagocyte respiratory bursting. Moreover, leukocytes isolated from murine and clinical CM generate higher ROS levels than those from healthy and non-CM controls [5, 6]. ROS/RNS were visualized in vivo using a sensitive probe, L-012, a luminol derivative with a higher sensitivity and signal to noise ratio than commonly used luminol and lucigenin [38, 39].

As suspected, these data show that mice with *P. berghei* infection are burdened with an increased ROS/RNS load throughout the body (Fig. 2). However, no emission signal was detected from the damaged brains, despite strong signals from damaged peripheral tissues (lungs, spleen, adipose). The absence of cerebral ROS/RNS signal through the development of fulminant, terminal ECM is intriguing. This finding is robust through two independent infections and a total of 27 mice in terminal-stage ECM (vehicle: n = 19, liraglutide: n = 8). Past experiments with this imaging molecule have shown penetration in the brain [35], including work in TBI where the damaged brain shows high levels of ROS/RNS [17].

The role of ROS/RNS in the pathogenesis of ECM is indeed unclear [8, 9, 11, 12, 40, 41]. Antioxidant treatments can be protective against ECM [41] and lipid peroxidation increased and cerebral thiols decreased in late-stage ECM [40, 42, 43]. Moreover, neutrophils accumulate in the ECM brain over time [44]. Finally, micro-haemorrhage sites in the cerebellum show signs of possible stress in the vicinity of the haemorrhage [45]. Conversely, antioxidant therapy was unsuccessful in treatment of human CM and hydrogen sulfide donors were ineffective against ECM [10, 37]. Indeed, many antioxidant treatments can also have effects on immune function [12, 46], which may partly explain their efficacy in ECM. Furthermore, although expression of some genes involved with oxidative stress is reduced in ECM [11], these seem to be compensated by increased anti-oxidant gene promotion [11, 47]: molecular markers of ROS damage (ROS-modified proteins) are largely unchanged [11, 12], mitochondrial function is preserved in ECM [8] and genetic removal of the primary endogenous reactive species generator NADPH oxidase does not improve ECM outcome [12]. Furthermore, poly(ADP-ribose) polymerase-1 knock-out mice are not protected from ECM, indicating that cytopathic hypoxia is not driving ECM [9]. These data are in support of these findings.

In a model of TBI, liraglutide reduced ROS/RNS and may have preserved mitochondrial function [17]. It is possible that an important component of the neuroprotective effect of GLP-1 involves the downstream buffering of cellular homeostasis and/or protection of mitochondrial function. These effects may be redundant in ECM and thus, be ineffective against fatality.

ROS/RNS were elevated in terminal mice in the lungs, spleen and adipose tissue- the preferred tissues where *P. berghei* parasites accumulated in previous studies [48, 49]. Interestingly, L-012 photon emission decreased in terminal mice and may reflect vascular collapse often associated with ECM vasospasms and increased RBC/iRBC/platelet/leukocyte aggregation in vessels throughout the body [50]. Therapeutic Epo treatment reduced reactive species in the lungs, spleen and adipose tissue. It is unclear whether this is a direct effect or may be associated with the reduced parasite load in Epo treated mice, similar to previous reports [31].

The CREB pathway is a well-described transcriptional pathway associated with the production of a wide range of protective proteins in the brain [51]. This appears to be the first assessment of CREB dynamics in the brains of ECM and NCM mice. It is clear from Fig. 3a, that the cerebral CREB system is activated specifically in ECM but not in healthy or NCM mice. Since CREB is involved in up-regulation of proteins with anti-oxidant properties and preservation of the mitochondria (e.g. haem oxygenase-1, peroxisome proliferator-activated receptor gamma coactivator 1-alpha, neuroglobin [52–54]), activation of this system in ECM may play a role in preventing ROS/RNS damage. Indeed, previous work by Linares et al. [11] shows that haem oxygenase-1 is markedly increased in mice with ECM and considered an important counterweight to potential oxidative damage. The activation of the CREB system observed here might be, in part, responsible for this mechanism.

In previous work, liraglutide treatment was protective after TBI and was associated with substantial pathology-dependent CREB activation and increases in CREB-regulated protective proteins [17]. Interestingly, CREB was not activated by liraglutide in ECM. This may be related to absence of ROS/RNS stress in the brains of ECM mice whereas ROS/RNS were markedly increased in TBI. Furthermore, it is intriguing that cAMP levels were increased in healthy mice by liraglutide but this did not translate to CREB activation. A similar phenomenon was observed previously, where liraglutide treatment did not activate CREB until pathology was introduced [17]. It seems that certain pathological events may be required for liraglutide to induce the CREB pathway. This work in ECM suggests that marked oxidative stress may be important for a therapeutic effect of GLP-1 treatment in the brain. Differences in the therapeutic effect of GLP-1 may also be related to changes in GLP-1 receptor expression during different pathological states. GLP-1 receptors are distributed in distinct brain regions in the healthy rodent brain [55]. Nevertheless, data is lacking on the receptor distribution under different pathological states. Finally, although GLP-1 treatment is known to increase the nitric oxide synthase enzyme, no increases in nitrous products downstream of nitric oxide production were detected in the brain. This may also contribute to the lack of effect of liraglutide in ECM, where nitric oxide is a known protective agent [20].

With an estimated 317 million people living with type II DM, a large portion of the burden resides in malaria endemic regions of the world [27]. It is therefore of interest to investigate the safety of type II DM treatments for potential interactions between a drug and parasite growth and development of malaria. These data show that *P. falciparum* growth in vitro and metabolism (Fig. 4) is unaffected by increasing doses of liraglutide (0.1–10 μg/mL). At a 100 μg/mL dosage, liraglutide treatment exerts inhibitory effects on parasite growth though is not likely to be achieved clinically due to gastrointestinal side effects associated with much lower doses [56, 57]. With three large in vivo experiments and in vitro experiments involving the human pathogen, it does seem likely that liraglutide interacts adversely with *P. falciparum* in humans. This investigation therefore suggests that liraglutide is likely safe for administration under co-morbidity circumstances with type II DM. Active post-marketing surveillance should still be applied.

## Conclusions

This shows that liraglutide, a neuroprotective drug in a wide-range of experimental models, is not effective against ECM. This may be related to the lack of ROS/RNS stress in the brain of ECM mice. Moreover, ROS/RNS may be adequately regulated by activation of the CREB system during ECM. This study may, thus, provide important insight into the pathogenesis of ECM and the mechanism driving the neuroprotective effects of GLP-1.

## Abbreviations

BBB: blood–brain barrier
CM: cerebral malaria
CREB: cyclic AMP response element binding
DM: diabetes mellitus
ECM: experimental cerebral malaria
Epo: erythropoietin
GLP-1: glucagon-like peptide-1
iRBC: infected erythrocyte
NCM: non-cerebral malaria
PA: Palo Alto
pCREB: phosphorylated_Ser133_ cyclic AMP response element binding
ROS/RNS: reactive oxygen/nitrogen species
TBI: traumatic brain injury
TBST: tris-buffered saline-tween20
