# Erratum to: Glucagon-like peptide-1 analogue, liraglutide, in experimental cerebral malaria: implications for the role of oxidative stress in cerebral malaria

**DOI:** 10.1186/s12936-016-1544-7

**Published:** 2016-09-27

**Authors:** Brian DellaValle, Casper Hempel, Trine Staalsoe, Flemming Fryd Johansen, Jørgen Anders Lindholm Kurtzhals

**Affiliations:** 1 Department of Immunology and Microbiology, Centre for Medical Parasitology, University of Copenhagen, Copenhagen, Denmark; 2 Department of Biomedical Sciences, Biotech Research and Innovation Center, Faculty of Health Sciences, University of Copenhagen, Copenhagen, Denmark; 3 Department of Clinical Microbiology, Copenhagen University Hospital, Copenhagen, Denmark

## Erratum to: Malar J (2016) 15:427 DOI 10.1186/s12936-016-1486-0

After publication of the original article [1], it came to the authors’ attention that there was an error in the final paragraph of the Discussion section. The word ‘not’ was accidently omitted from the following sentence: “With three large in vivo experiments and in vitro experiments involving the human pathogen, it does seem likely that liraglutide interacts adversely with *P. falciparum* in humans.”

The sentence should therefore have read as follows: “With three large in vivo experiments and in vitro experiments involving the human pathogen, it does **not** seem likely that liraglutide interacts adversely with *P. falciparum* in humans.”
